# Electroencephalogram-Based Brain Connectivity Analysis in Prolonged Disorders of Consciousness

**DOI:** 10.1155/2023/4142053

**Published:** 2023-04-18

**Authors:** Yuzhang Wu, Zhitao Li, Ruowei Qu, Yangang Wang, Zhongzhen Li, Le Wang, Guangrui Zhao, Keke Feng, Yifeng Cheng, Shaoya Yin

**Affiliations:** ^1^Clinical College of Neurology, Neurosurgery, and Neurorehabilitation, Tianjin Medical University, Tianjin 300000, China; ^2^Department of Neurosurgery, Tianjin Huanhu Hospital, Tianjin 300000, China; ^3^State Key Laboratory of Reliability and Intelligence of Electrical Equipment, Hebei University of Technology, Tianjin 300000, China

## Abstract

**Background:**

Prolonged disorders of consciousness (pDOC) are common in neurology and place a heavy burden on families and society. This study is aimed at investigating the characteristics of brain connectivity in patients with pDOC based on quantitative EEG (qEEG) and extending a new direction for the evaluation of pDOC.

**Methods:**

Participants were divided into a control group (CG) and a DOC group by the presence or absence of pDOC. Participants underwent magnetic resonance imaging (MRI) T1 three-dimensional magnetization with a prepared rapid acquisition gradient echo (3D-T1-MPRAGE) sequence, and video EEG data were collected. After calculating the power spectrum by EEG data analysis tool, DTABR ((*δ* + *θ*)/(*α* + *β*) ratio), Pearson's correlation coefficient (Pearson *r*), Granger's causality, and phase transfer entropy (PTE), we performed statistical analysis between two groups. Finally, receiver operating characteristic (ROC) curves of connectivity metrics were made.

**Results:**

The proportion of power in frontal, central, parietal, and temporal regions in the DOC group was lower than that in the CG. The percentage of delta power in the DOC group was significantly higher than that in the CG, the DTABR in the DOC group was higher than that in the CG, and the value was inverted. The Pearson *r* of the DOC group was higher than that of CG. The Pearson *r* of the delta band (*Z* = −6.71, *P* < 0.01), theta band (*Z* = −15.06, *P* < 0.01), and alpha band (*Z* = −28.45, *P* < 0.01) were statistically significant. Granger causality showed that the intensity of directed connections between the two hemispheres in the DOC group at the same threshold was significantly reduced (*Z* = −82.43, *P* < 0.01). The PTE of each frequency band in the DOC group was lower than that in the CG. The PTE of the delta band (*Z* = −42.68, *P* < 0.01), theta band (*Z* = −56.79, *P* < 0.01), the alpha band (*Z* = −35.11, *P* < 0.01), and beta band (*Z* = −63.74, *P* < 0.01) had statistical significance.

**Conclusion:**

Brain connectivity analysis based on EEG has the advantages of being noninvasive, convenient, and bedside. The Pearson *r* of DTABR, delta, theta, and alpha bands, Granger's causality, and PTE of the delta, theta, alpha, and beta bands can be used as biological markers to distinguish between pDOC and healthy people, especially when behavior evaluation is difficult or ambiguous; it can supplement clinical diagnosis.

## 1. Introduction

pDOC refers to the disorder of consciousness that has lost consciousness for more than 28 days [1]. pDOC is divided into vegetative state (VS) and minimally conscious state (MCS). VS, also known as unresponsive awakening syndrome (UWS), refers to the existence of basic brainstem reflexes and sleep-wake cycles, without conscious content [2]. MCS refers to discontinuous and fluctuating consciousness signs of patients [3]. In recent years, with the progress of medical technology, it is found that some pDOC patients show signs of brain activity, indicating that their brains have hidden consciousness. Therefore, some new diagnostic classifications have been proposed, such as “cognitive motor dissociation,” “hidden cortical activity,” and “MCS ^∗^.” [4–6]. Assessment is an important part of pDOC management, which determines what treatment patients should receive next because the therapy of VS and MCS are completely different [3]. At present, the commonly used clinical evaluation methods are as follows: (1) coma recovery scale-revise (CRS-R); (2) neuroimaging; (3) neuroelectrophysiology.

The CRS-R is the “gold standard” of behavioral assessment. It consists of 6 subscales, involving auditory, verbal, visual, communication, motor, and arousal levels, including 23 hierarchical and orderly scoring criteria. However, many patients with pDOC cannot produce purposeful behavior activities due to movement disorders, which may be misdiagnosed. It is estimated that about 15% of patients who meet the VS behavioral criteria have cognitive motor dissociation or covert consciousness, which can only be found through neuroimaging or other technical methods [7]. Theoretically, consciousness is mainly the subjective experience of the subject itself, which is not equal to the explicit behavior expression of the subject. For pDOC like VS, the fluctuation of motor dysfunction and arousal level makes the misdiagnosis rate of VS as high as 40% [8]. As we study the neural network hidden behind consciousness, more and more evidence shows that when diagnosing pDOC, we should use neuroelectrophysiology and neuroimaging methods to make a precise diagnosis [9].

Neuroimaging and neuroelectrophysiology examinations are not limited to diagnostic purposes. They can provide a prognostic reference, surrogate markers of therapeutic efficacy, and clinical evaluation. At present, computed tomography (CT), MRI, fluorodeoxyglucose positron emission computed tomography (FDG-PET), and functional near-infrared spectroscopy (fNIRS) are commonly used in clinical neuroimaging. CT is mainly used to diagnose cerebral hemorrhage and brain trauma. Structural MRI can provide a three-dimensional high-resolution characterization of gray matter and white matter changes after brain injury. The diffusion-weighted image (DWI) results show that the apparent diffusion coefficient (ADC) is a useful auxiliary index to predict the neural prognosis of patients with cardiac arrest [10]. Resting-state functional MRI (r-fMRI) uses blood oxygen level dependent (BOLD) to display the resting state image of the brain and can generate a statistical graph of the whole brain connection of a specific region or network by associating the BOLD signal in the region of interest (ROI) with all other voxels in the brain [11]. In addition, fMRI can draw brain network components closely related to consciousness, such as default mode network (DMN), salience network (SN), and executive control network (ECN), and serve as neural imaging biomarkers for pDOC prognosis [12, 13]. But MRI also has its limitations, for example, the machine cannot be moved; it is not suitable for metal implants; it requires a supine position, and there are motion artifacts. FDG-PET reflects metabolism by measuring the amount of glucose absorbed by brain tissue. FDG-PET has high sensitivity and specificity to distinguish patients with pDOC, and it can distinguish patients with VS and MCS [9]. FDG-PET can detect patients with relatively reserved brain metabolism early, reduce misdiagnosis rate, and risk of premature termination of life support treatment [14]. Its disadvantages are radioactive and expensive. fNIRS uses near-infrared light to detect the concentration changes of oxyhemoglobin and deoxyhemoglobin in blood. Therefore, fNIRS, like BOLD, is an indirect imaging tool. Molteni et al. conducted sensory and motor stimulation on MCS patients using fNIRS equipment and found that somatosensory stimulation caused weak hemodynamic responses in the sensory cortex, but passive motion stimulation produced clearer hemodynamic responses, and active motion tasks produced weak hemodynamic responses in the hand area of M1 [15]. Another study found that compared with patients in VS, patients in MCS often showed similar hemodynamic responses to healthy people [16]. However, this technology is still mostly used in scientific research in China and has not been widely used in clinical practice.

EEG is a noninvasive, bedside, mobile examination. Its principle is to amplify and record the spontaneous biological potential of the brain from the scalp, which is the spontaneous and rhythmic electrical activity of the brain cell group recorded by electrodes. For pDOC patients, resting EEG is the main method in clinical evaluation. EEG can be used to assess the integrity of the sleep-wake cycle of patients [17], and studies have found that the existence of sleep spindles is related to covert consciousness [18]. There is no systematic change in sleep spindle wave and slow wave oscillation between daytime and nighttime in patients with pDOC [19]. Event-related potential (ERP) is a kind of special evoked potential when given a specific stimulus to the nervous system or the brain processes the stimulus information, which refers to the bioelectrical response that can be detected in the corresponding parts of the system and the brain and is related to the timing and specific location of the stimulus, generally based on EEG acquisition. At present, P300 and mismatch negativity (MMN) are the most widely used. P300 is a positive wave that appears about 300 ms after stimulation caused by oddball paradigms. It can be found in a large number of healthy people, MCS, and locked-in syndrome (LIS), but it is rare in VS [20]. P300 reflects the brain's ability to process information. The amplitude of P300 was related to the CRS-R score, and the latency of P300 in pDOC patients was prolonged [21–23]. MMN is the difference wave obtained by averaging the ERP of the standard stimulus and deviation stimulus, respectively, subtracting the ERP of the standard stimulus from the ERP of the deviation stimulus. The negative deflection of 100~250 ms after the difference of stimulus is MMN [24]. MMN can distinguish between healthy patients and DOC patients, and MMN is often low in VS [25, 26]. Spectrum power, functional connection, dynamic functional connectivity, graph theory, microstate, and nonlinear measurement of EEG are also widely used in the research of pDOC [20]. EEG is similar to fMRI, data can be used to build a multiscale brain network composed of nodes (hub) and their connections, in which topology can be quantified. Some studies have proved the usefulness of complex brain network analysis for pDOC patients by extracting the resting state EEG index and can distinguish MCS and VS [27, 28]. The local and overall efficiency of the resting state brain network of pDOC patients is reduced, and the hub of the alpha band is reduced [29]. qEEG, based on the classical EEG, recording the electrophysiological activities of brain neurons, transforms the original, complex, and changeable electrophysiological curve into quantitative, single, and orderly natural data, thus improving the sensitivity to changes in brain function [30]. At present, many qEEG indicators with prognostic potential have been found. For example, the DTABR can predict the prognosis of patients with pDOC [31]. Among various brain connectivity measurement methods, EEG-based brain connectivity uniquely provides millisecond resolution. There are many methods to analyze brain connectivity through EEG, some of which estimate synchronization of distributed EEG signals recorded from the head surface, and some of which estimation of functional connectivity from the surface Laplacian, but these methods cannot completely eliminate the impact of volume conductance, thus affecting the results [32]. The EEG source analysis can reduce the volume conduction effect and overcome these limitations. Pearson *r*, Granger's causality, and PTE have commonly used indexes based on EEG brain connection. Pearson *r*, also known as Pearson's product-moment correlation coefficient, is a linear correlation coefficient, which reflects the linear correlation degree of two variables, ranging from -1 to 1. The larger the absolute value, the stronger the correlation. Granger's causality is a method of functional connectivity, which was introduced by Clive W.J. Granger and later improved by Geweke in the form used today. [33] Specifically, Granger's causality is a useful tool to define the cause and effect of neurophysiological time series [34]. PTE is a new oriented connectivity measurement method based on information theory. It has many advantages and is particularly suitable for connectivity analysis of EEG data [35].

At present, there is a certain misdiagnosis rate in the clinical behavioral evaluation of pDOC. The purpose of this study is to determine the difference between the brain connectivity of pDOC patients and healthy people, explore the characteristics of the brain connectivity of pDOC patients, and evaluate whether EEG-based brain connectivity can be used as a significant biomarker in the diagnosis of pDOC. This study uses the EEG source analysis and has a strict data screening process, so our results may reflect objective authenticity.

## 2. Materials and Methods

### 2.1. Participants

**Figure 1:**
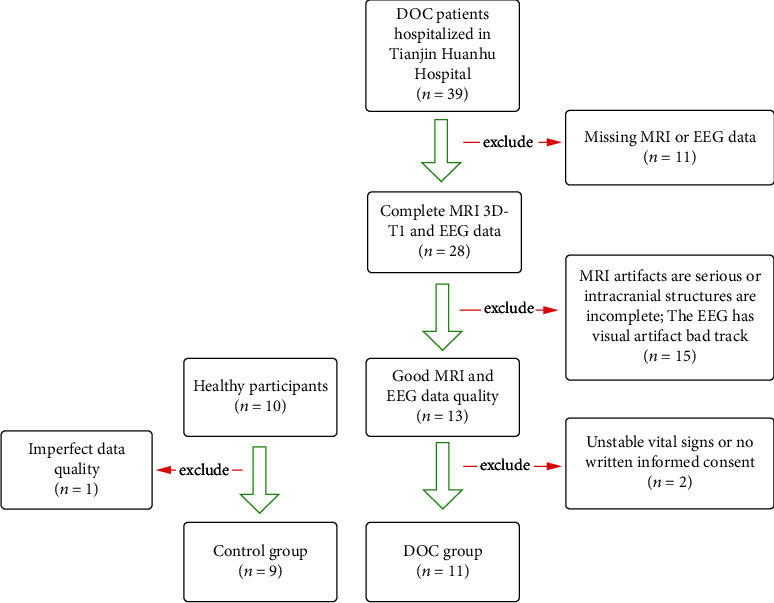
The CONSORT diagram.

pDOC patients who were treated and evaluated in the neurosurgery department of Tianjin Huanhu Hospital from January 2021 to October 2022 were included. Inclusive criteria are as follows: (1) disorder of consciousness greater than 28 days; (2) after admission, professional doctors should complete more than three CRS-R scores; (3) video EEG monitoring is more than 12 hours; (4) complete the head MRI examination; (5) the intracranial structure is relatively complete and without skull defect; (6) complete clinical data. The exclusion criteria are as follows: (1) the data quality is poor, and there are many artifacts; (2) use sedation or anesthetics during data acquisition; (3) unstable vital signs. This study was approved by the Medical Ethics Committee of Tianjin Huanhu Hospital and obtained the written informed consent of all participants or legal guardians. At the same time, collected the resting EEG and MRI data of 9 healthy participants (Figure 1).

### 2.2. Data Acquisition

General data of pDOC patients were collected, including age, sex, primary disease, brain injury regions, and admission CRS-R score. The age and sex data of the CG were collected. SIEMENS SKYRA 3 T was used for MRI and collected 3D-T1-MPRAGE sequence. MRI parameters are as follows: repetition time (TR) = 2000 ms, echo time (TE) = 3.0 ms, and slice thickness = 1 mm. Turning Angle = 9°, Slice Gap: 50 percent, Resolution Matrix = 256 × 256. The rest-state scanning lasts for 4 minutes and 40 seconds. The video EEG signal was sampled with the Nutricle medical event-related potentiometers. We use a 32-channel cap with 20 active electrodes, as done in the clinical practice. The electrode position was based on the 10-20 system, the sampling rate was 2 kHz, and the band-pass filter was 0.1 Hz to 250 Hz. The impedance of all recording electrodes was kept below 10 k*Ω*. EEG data were recorded with medical event-related potentiometers software (Nuclear, China). In order to minimize the result error, EEG analysis was then performed on an awake EEG signal. [36] Each healthy participant was in a quiet room. During the data collection, they were asked to open their eyes and rest and collected EEG data for 15 minutes. The EEG selected by each pDOC participant is the EEG of eye-opening. pDOC and healthy participants performed the same EEG protocol.

### 2.3. EEG Analysis

EEG indicators use MATLAB 2020b (Mathworks Inc., Natick, Massachusetts, United States) Brainstorm toolbox (https://neuroimage.usc.edu/brainstorm) to calculate. EEG preprocessing was carried out first. 50 Hz alternating current interference wave was removed by a notch filter and performed 0.5 Hz-40 Hz band-pass filtering. Then, independent component analysis (ICA) was performed by the Picard method to eliminate heartbeat and blink artifacts. The MRI 3D-T1 sequence data was imported, and the EEG electrode was positioned on the 3D-T1 image. EEG electrodes are divided into five regions: frontal region (Fp1, Fp2, F3, Fz, F4, F7, and F8), central region (C3, Cz, and C4), temporal region (T3, T4, T5, and T6), parietal region (P3, Pz, and P4), and occipital region (O1, Oz, and O2). 1-3 Hz is the delta band, 4-7 Hz is the theta band, 8-13 Hz is the alpha band, and 14-30 Hz is the beta band. The frontal region power is the sum of the frontal region electrodes' power; using the Welch method, calculate the power spectrum and calculate the power of the central region, temporal region, parietal region, and occipital region in turn. The total power is defined as the sum of all electrodes' power. The ratio of the power of each zone and each frequency band to the total power is the power ratio. Calculate DTABR at the same time. The Freesurfer toolbox (https://surfer.nmr.mgh.harvard.edu) was used to conduct comprehensive segmentation and surface reconstruction of structural MRI, forming a high-definition cortical layer and boundary surface of the brain, skull, and scalp. These surfaces were then used to construct a boundary element method (BEM) model. The conductivity value is assigned to each interval. The standard contour of the electrode position is digitized and registered with the reference point on the template brain. The high-density cortical mesh is used as the source space. Then, the standardized low-resolution brain electromagnetic tomography (sLORETA) is used to locate the source of EEG signals. The Desikan-Killiany brain atlas [37] was used for our research. Calculate the Pearson *r* and generate the corresponding matrix graph. Calculate the binary Granger causality, the maximum order of using the Granger model is 6, and generate the matrix diagram and circos diagram. Finally, calculate PTE. 1-3 Hz is the delta band, 4-7 Hz is the theta band, 8-13 Hz is the alpha band, 14-30 Hz is the beta band, and generate the PTE matrix diagram and corresponding connection diagram.

### 2.4. Statistical Analysis

All statistical calculations were performed in SPSS 26.0. The measurement data conforming to the normal distribution are expressed in mean ± SD, and the classified variables are reported as numbers (*n*) and proportions (%). The nonparametric test is adopted for the comparison of measurement data components with the abnormal distribution. The ROC of the connectivity index was made and the area under the curve (AUC) was calculated. *P* < 0.05 was statistically significant.

## 3. Results and Discussion

### 3.1. Participant Characteristics

There were 9 healthy participants in CG, with an average age of 29.1 ± 8.0 years, including 5 (55.6%) males and 4 (44.4%) females. There were 11 participants in the DOC group, with an average age of 36.9 ± 16.3 years, there are 5 (45.5%) males and 6 (54.5%) females. The causes of the disease were cerebral hemorrhage in 7 (63.6%) and brain injury in 4 (36.4%). There were 5 (45.5%) participants with VS, 5 (45.5%) participants with MCS, and 1 (9.0%) participant with MCS. The clinical data of the DOC group are shown in Table 1.

### 3.2. Power Spectrum

**Figure 2:**
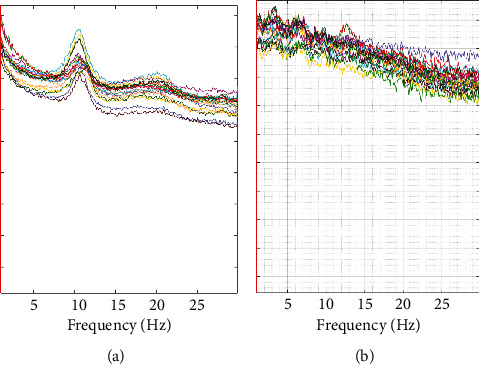
Comparison of power spectrum between DOC group and CG group. (a) Power spectrum of the CG group, higher power can be seen in the alpha band of 10-15 Hz. (b) The power spectrum of the DOC group. It is dominated by high slow wave power. (DOC: disorder of consciousness; CG: control group).

**Figure 3:**
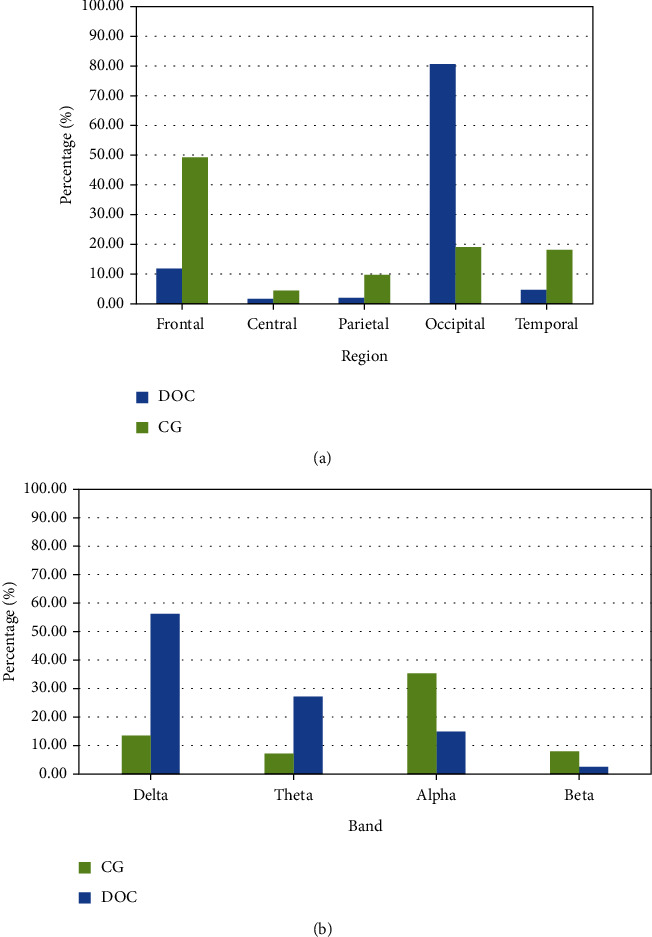
Bar chart of relative power comparison. (a) Area power bar chart. The power ratio of frontal area (Fp1, Fp2, F3, Fz, F4, F7, and F8), central area (C3, Cz, and C4), temporal area (T3, T4, T5, and T6), parietal area (P3, Pz, and P4), and occipital area (O1, Oz, and O2). In the DOC group, the power ratios of frontal, central, parietal, and temporal regions decreased significantly. (b) Area power bar chart. It can be seen that the proportion of power in delta and theta bands in the DOC group is significantly higher than that in the CG group, while the proportion of power in alpha and beta bands in the CG group is higher than that in the DOC group, this may be the cause of the DTABR difference. (DOC: disorder of consciousness; CG: control group).

We calculated the power spectrum of 11 participants in the DOC group and 9 participants in CG who met the inclusion criteria (Figure 2). In the DOC group, the frontal power accounted for 11.68% of the total power, and the central, parietal, occipital, and temporal power accounted for 1.43%, 1.69%, 80.79%, and 4.40% of the total power, respectively. In the DOC group, the delta band power accounts for 56.02% of the total power, and the power in theta, alpha, and beta bands accounts for 27.11%, 14.61%, and 2.25% of the total power, respectively. DTABR is 4.93 (Figure 3). In the healthy group, the frontal power accounted for 49.24% of the total power, while the central, parietal, occipital, and temporal power accounted for 4.16%, 9.47%, 19.01%, and 18.11% of the total power, respectively. In the healthy group, the delta power accounted for 13.34% of the total power, and the power in theta, alpha, and beta bands accounted for 6.87%, 35.10%, and 7.72% of the total power, respectively. DTABR is 0.47 (Figure 3).

### 3.3. Correlation Indicators

**Figure 4:**
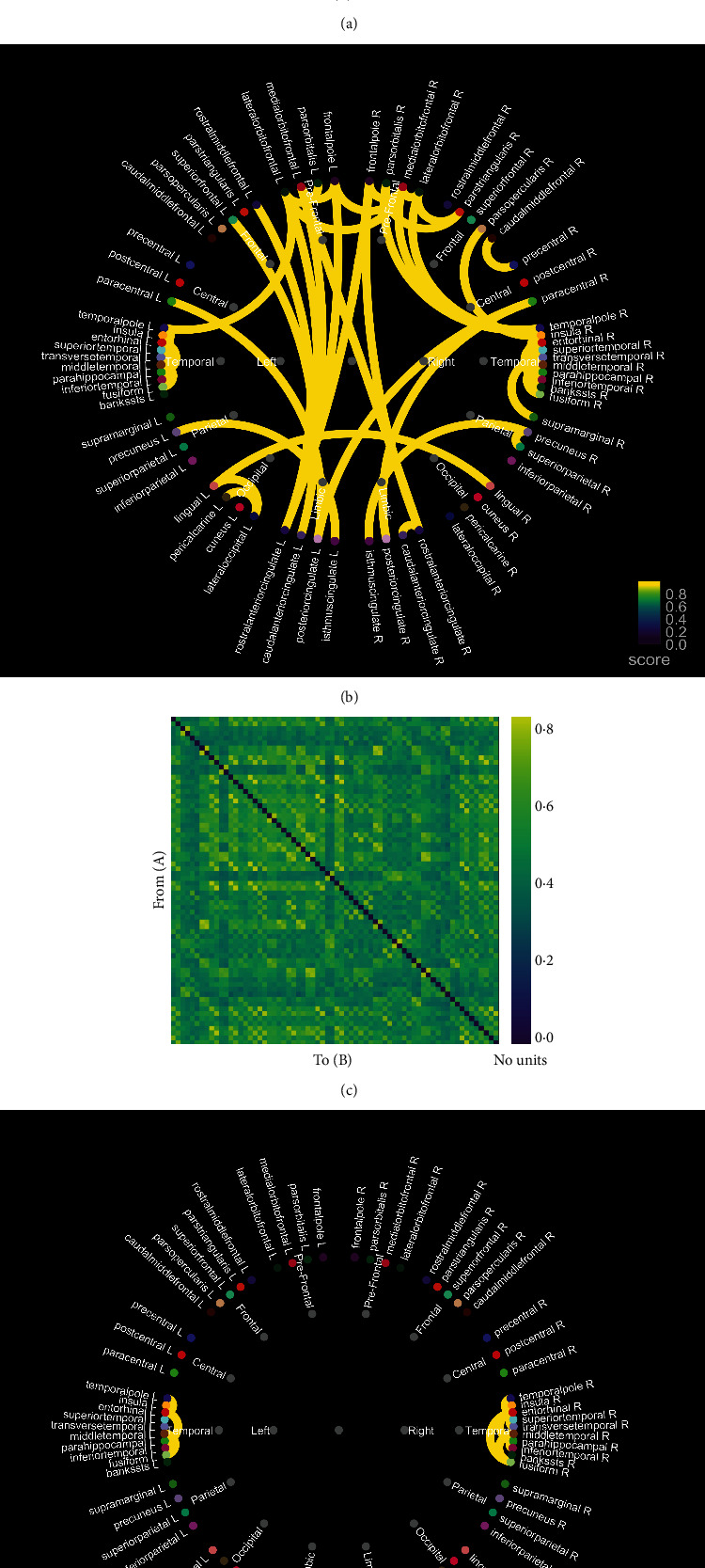
The Pearson *r* matrix diagram and circos diagram. (a, b) CG group connection matrix diagram. It can be seen that the Pearson *r* is higher in each brain region and between hemispheres. (c, d) In the DOC group, only a few connections were found in the temporal lobe and no obvious connection between hemispheres. (DOC: disorder of consciousness; CG: control group).

**Figure 5:**
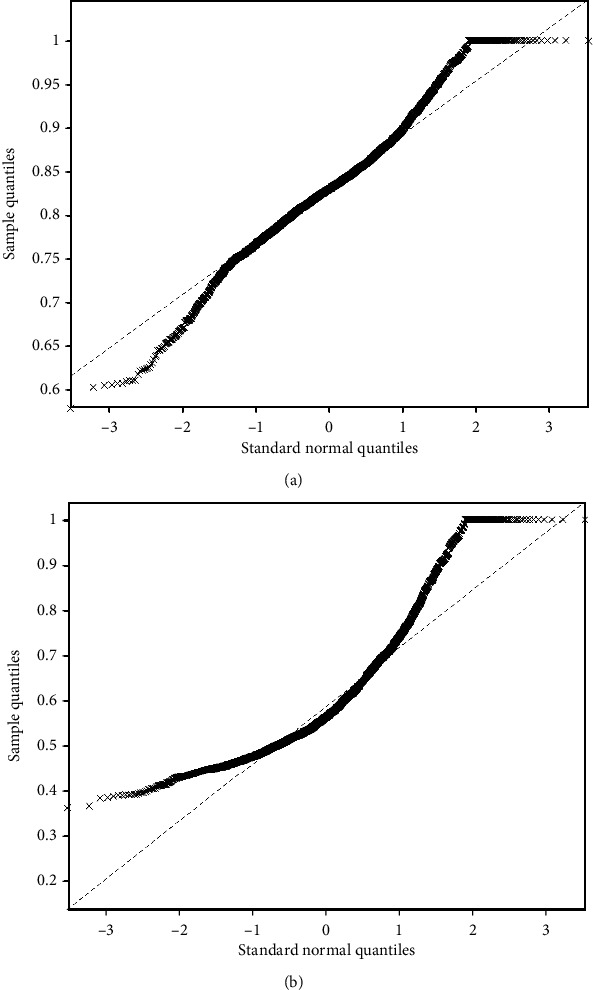
The Pearson *r* normal distribution quantitative plot (Q-Q plot). (a) CG group Q-Q plot. The data is the normal distribution. (b) DOC Q-Q plot. The data showed a left-skewed distribution.

**Figure 6:**
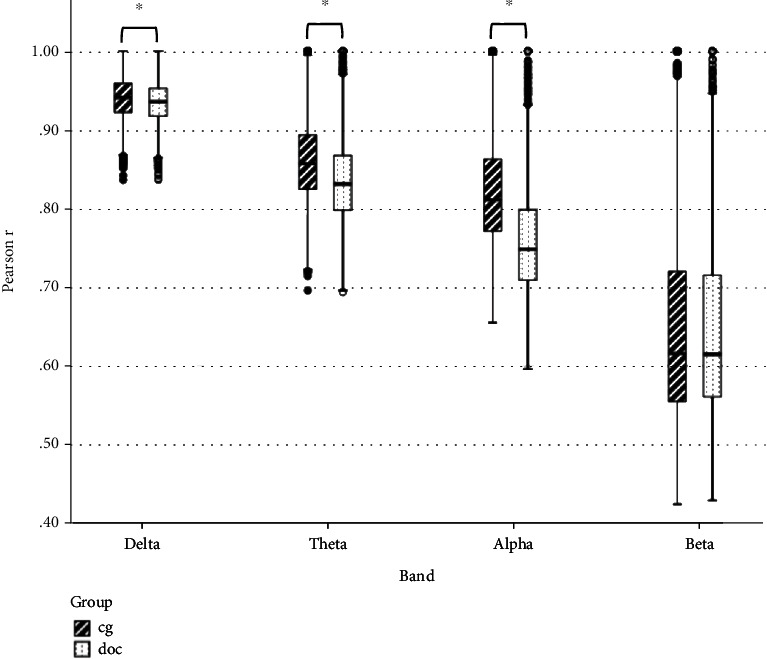
The Pearson *r* results using Mann–Whitney *U* test. Delta, theta, and alpha bands are statistically significant. (^∗^ represents *P* < 0.05).

**Figure 7:**
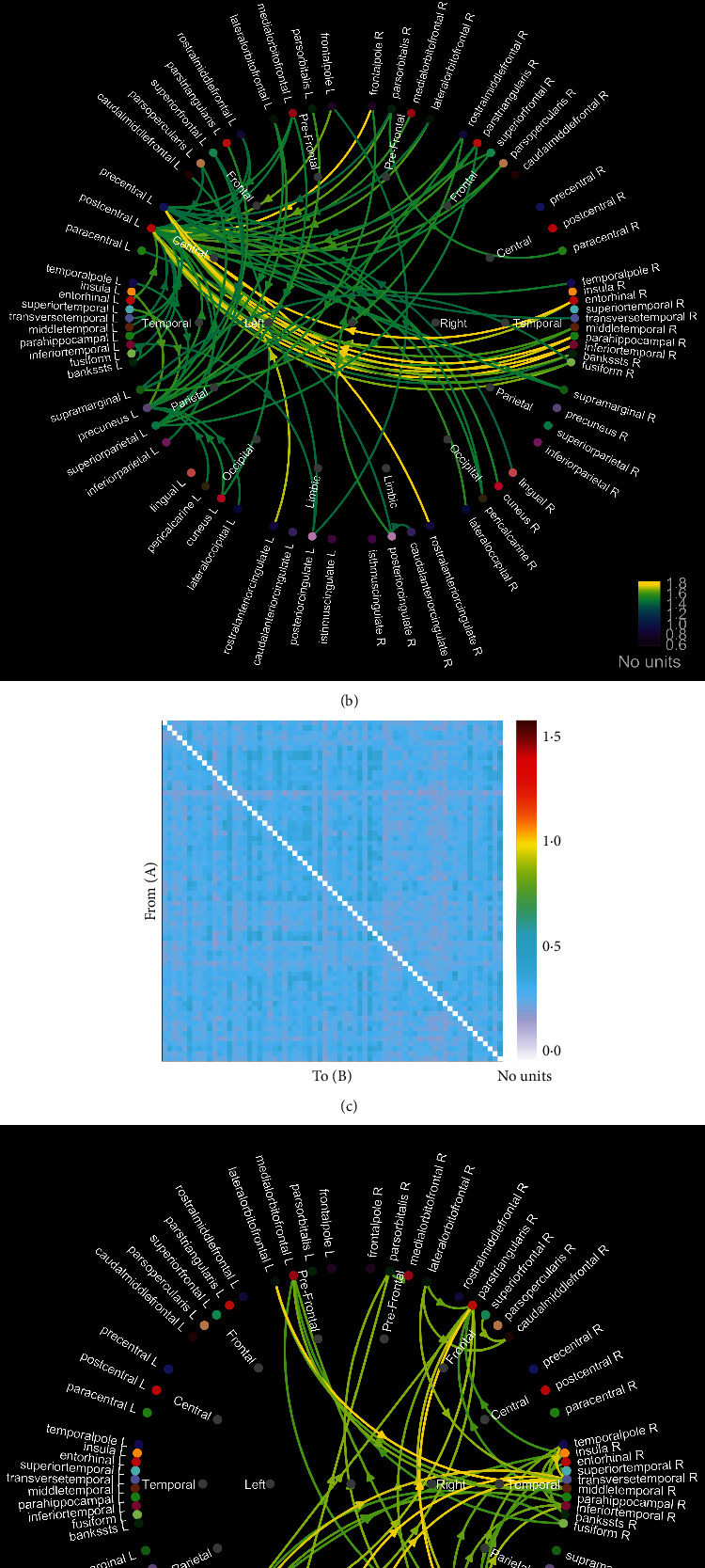
Granger's causality matrix diagram and circos diagram. (a, b) The Granger causality of the CG group, with more directional connections. (c, d) The Granger causality of DOC group. At the same threshold, the intensity of directed connections in the DOC group decreased significantly, and the number and intensity of directed connections between hemispheres in the DOC group decreased compared with that in the CG group. (DOC: disorder of consciousness; CG: control group).

**Figure 8:**
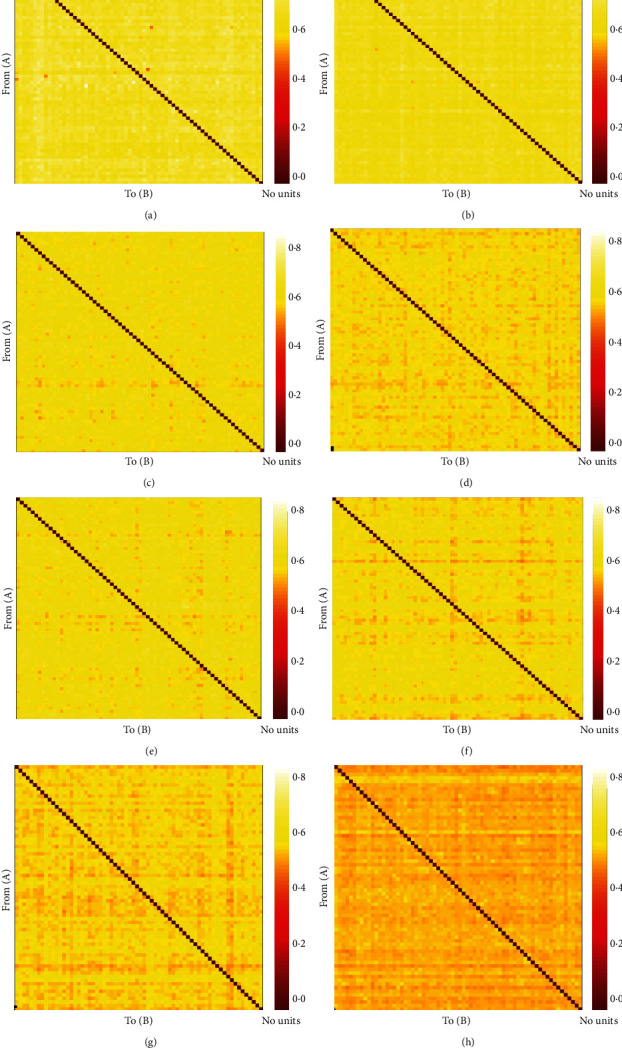
PTE matrix diagram and circos diagram. (a, b) Delta band PTE. (c, d) Theta band PTE. (e, f) Alpha band PTE. (g, h) Beta band PTE. PTE of delta band (*Z* = −42.68, *P* < 0.01), theta band (*Z* = −56.79, *P* < 0.01), alpha band (*Z* = −35.11, *P* < 0.01), and beta band (*Z* = −63.74, *P* < 0.01) is statistically significant. (PTE: phase transfer entropy).

**Figure 9:**
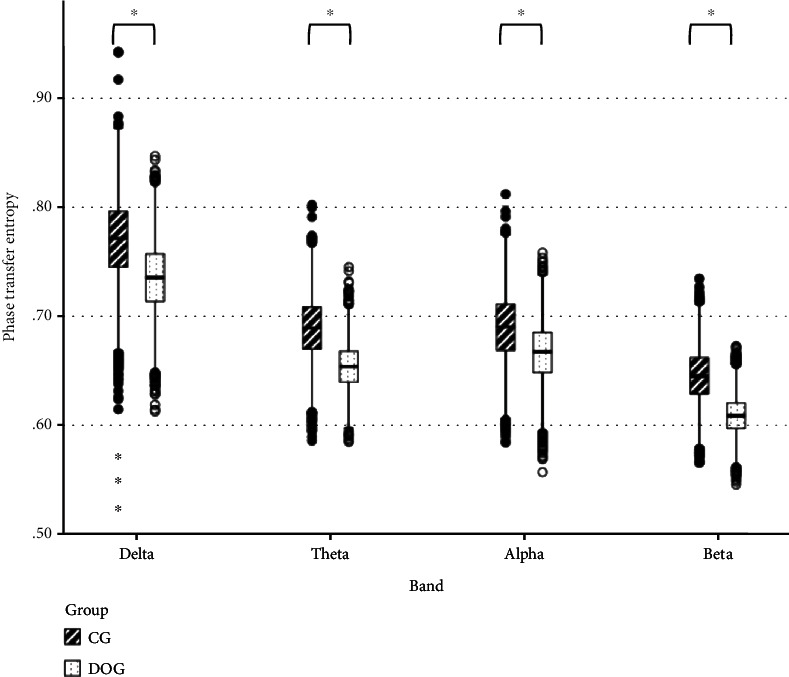
PTE results using Mann–Whitney *U* test. Delta, theta, alpha, and beta bands are statistically significant. (^∗^ represents *P* < 0.05) (PTE: phase transfer entropy).

The Pearson *r* matrix diagram and circos diagram are shown in Figure 4, which shows that Pearson *r* in the DOC group is higher than that in CG. The quantile-quantile plot (Q-Q plot) shows that the Pearson *r* data of the DOC group and the CG do not conform to the normal distribution (Figure 5), so the Mann–Whitney *U* test is used for the statistical between groups. Among them, the Pearson *r* of delta band (*Z* = −6.71, *P* < 0.01), theta band (*Z* = −15.06, *P* < 0.01), and alpha band (*Z* = −28.45, *P* < 0.01) is statistically significant in distinguishing DOC and healthy people (Figure 6). Granger's causality matrix is shown in Figure 7. Mann–Whitney U test of Granger's causality was statistically significant (*Z* = −82.43, *P* < 0.01). At the same threshold, the intensity of directed connections in the DOC group decreased significantly, and the number and intensity of directed connections between hemispheres in the DOC group decreased compared with that in CG. The information flow in the CG mostly concentrated in the central area and parietal lobe, while the information in the DOC group mostly flowed to the temporal lobe. The matrix diagram of each frequency band of PTE was shown in Figure 8. It can be seen that the frequency bands of PTE in the DOC group are lower than those in CG. The data of PTE is abnormal distribution, so the Mann–Whitney *U* test was used for statistics between groups, in which the delta band (*Z* = −42.68, *P* < 0.01), theta band (*Z* = −56.79, *P* < 0.01), the alpha band (*Z* = −35.11, *P* < 0.01), and beta band (*Z* = −63.74, *P* < 0.01) had statistical significance in distinguishing DOC (Figure 9).

**Figure 10:**
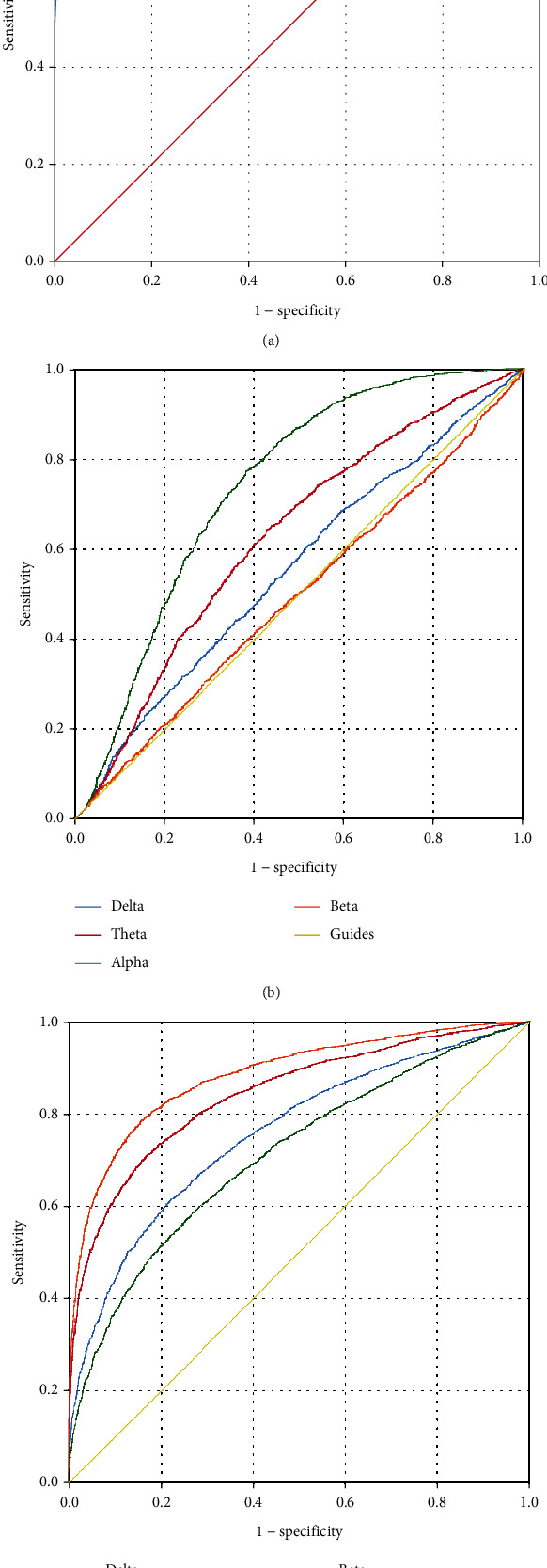
ROC curves for different connectivity metrics. (a) ROC curve of Granger's causality. AUC = 0.0995 (95% CI 0.004-0.996 *P* < 0.01). (b) ROC curve of the Pearson *r*. Delta AUC = 0.557 (95% CI 0.540-0.573 *P* < 0.01), theta AUC = 0.627 (95% CI 0.611-0.643 *P* < 0.01), alpha AUC = 0.740 (95% CI 0.726-0.754 *P* < 0.01), and beta AUC = 0.497 (95% CI 0.480-0.513 *P* = 0.71). (c) ROC curve of PTE. Delta AUC = 0.756 (95% CI 0.746-0.766 *P* < 0.01), theta AUC = 0.841 (95% CI 0.833-0.849 *P* < 0.01), alpha AUC = 0.711 (95% CI 0.700-0.721 *P* < 0.01), and beta AUC = 0.883 (95% CI 0.876-0.890 *P* < 0.01). (ROC: receiver operating characteristic curve; AUC: area under curve; PTE: phase transfer entropy).

The ROC of connectivity indicators shows (Figure 10) that the ROC of PTE AUC = 0.0995 (95% CI 0.004-0.996 *P* < 0.01); The ROC of the Pearson *r* delta band AUC = 0.557 (95% CI 0.540-0.573 *P* < 0.01), theta band AUC = 0.627 (95% CI 0.611-0.643 *P* < 0.01), alpha band AUC = 0.740 (95% CI 0.726-0.754 *P* < 0.01), beta band AUC = 0.497 (95% CI 0.480-0.513 *P* = 0.71); The PTE ROC of delta band AUC = 0.756 (95% CI 0.746-0.766 *P* < 0.01), theta band AUC = 0.841 (95% CI 0.833-0.849 *P* < 0.01), alpha band AUC = 0.711 (95% CI 0.700-0.721 *P* < 0.01), and beta band AUC = 0.883 (95% CI 0.876-0.890 *P* < 0.01).

### 3.4. Discussion

The purpose of this research is to explore the brain functional connectivity difference between pDOC patients and healthy ones. We strictly screen data, use EEG with good quality, eliminate errors caused by data screening, and use the source EEG analysis to calculate the power spectrum, the Pearson *r*, Granger's causality, and PTE to evaluate connectivity. We only used bedside EEG and MRI 3D-T1 sequences. Therefore, it provides a quantitative evaluation of the selectivity of patients with pDOC for grassroots hospitals and a supplement for behavioral evaluation. In order to minimize the result error, we choose the awake EEG with eyes open, because the pDOC patients cannot cooperate with our instructions, and we cannot determine whether the pDOC patients are in a state of meditation. They may concentrate on thinking, falling asleep, or any other situation.

In this study, we calculated the regional power ratio, frequency band power ratio, and DTABR. The absolute total power seems to have nothing to do with the classification of pDOC but is related to the etiology. It is usually lower in patients with hypoxic-ischemic encephalopathy, but it is impossible to distinguish TBI from stroke [38]. In the regional power ratio, it is worth noting that the power ratio of the frontal region is the most obvious in pDOC patients. At the same time, the percentage of occipital power in the pDOC group increased significantly. Our statistics found that the absolute value of occipital power decreased, but the percentage increased. It is considered that the decrease in occipital power is not as obvious as that in other regions. In the frequency band proportion, the CG has the highest proportion of alpha band, while the DOC group has the highest proportion of delta band. The function of the prefrontal lobe has been found more and more at present. The prefrontal lobe is an important center of consciousness and the content of consciousness. It can regulate thalamic activity and affect consciousness [39]. The change of frontal lobe power in this study seems to prove the important role of the frontal lobe in consciousness. Research shows that the alpha band is believed to come from the IV and V layers of the cortex [40], so the decrease of alpha band power proportion in pDOC patients may be the cause of cortical functional damage. Alpha band power is positively correlated with regional cerebral blood flow and oxygen metabolism [41]. Cortical atrophy in pDOC patients may also be one of the reasons for the decrease of alpha band power. The pDOC patients' EEG usually shows that the background activity slows down, and the visual EEG analysis shows that this background slowing is closely related to the loss and reduction of alpha wave [42]. The closer the alpha band power is to healthy people, the greater the probability of consciousness recovery [20]. Several studies have found that the delta band power is negatively correlated with the CRS-R score, that is, the VS has a higher delta band power than the MCS [29, 43]. Lutkenhoff et al. used MRI and found that the higher the degree of bilateral thalamus and globus pallidus atrophy, the lower the relative power [36]. The atrophy of the brain stem left globus pallidus and right caudate nucleus was more extensive in patients with lower total power [36]. DTABR is the ratio of the slow wave to the fast wave and can evaluate the evaluation of pDOC and the prognosis of pDOC. It is an ideal indicator to reflect brain injury and disorder of consciousness [44]. We found that the DTABR of the DOC group was significantly lower than that of the CG, and the value is inversion.

Functional connectivity measures the statistical dependence of electrophysiology time series recorded in different brain regions. Because the calculation of functional connectivity highly depends on the changes in brain activity in time series, high-time resolution techniques such as EEG (<1 ms) are the best choice to reflect neural dynamics and rapid response. We found that the Pearson *r* of the delta, theta, and alpha bands were significantly different between DOC and CG. These results are similar to some research results, and the level of connectivity is related to the severity of pDOC [44]. In the Granger causality of this study, the number and intensity of directed connections in pDOC patients decreased significantly. A Granger causality study on neurocognitive dysfunction and disorder of consciousness shows that in the pDOC group, all frequency band connections are reduced, and there are also significant differences in the connections from other cortical regions to frontal lobe regions [45], which seems to further indicate the role of the frontal lobe in consciousness. We also found that the directional connection between the hemispheres of pDOC is reduced, and the information communication seen in the cerebral hemisphere seems to be a sign of the existence of consciousness, which needs further research to prove. At present, transfer entropy is still controversial for distinguishing VS, MCS, eMCS (escape MCS), and healthy people [46], but some studies show that transfer entropy, especially in the alpha band, can distinguish VS and MCS [47]. We found that PTE was significantly different in the four frequency bands of the two groups, so we thought that the four frequency bands of PTE could distinguish the DOC group and CG.

### 3.5. Limitations

This study has some limitations. Age is one of the factors that cannot be ignored in EEG connectivity analysis, and the brain connectivity of the elderly and young may be different [48]. The average age of subjects in the DOC group was slightly higher than that in the control group. This study is still in the early stage of work, and the number of participants included is small. The DOC group could not be subdivided according to the level of consciousness. A large-scale statistical study will be conducted after a large number of cases in the future.

## 4. Conclusions

Brain connectivity analysis based on EEG has the advantages of being noninvasive, convenient, and bedside. It does not require special equipment and can be carried out in most grassroots hospitals. The DTABR, delta, the Pearson *r* of theta and alpha bands, Granger's causality, and PTE of the delta, theta, alpha, and beta bands calculated by qEEG can be used as biological markers to distinguish pDOC and healthy people, especially in the case of difficult or ambiguous behavior evaluation, to supplement clinical diagnosis.

## Figures and Tables

**Table 1:** Clinical data of participants in the DOC group.

	Sex	Age	Pathogenesis	Conscious	Conscious of follow up
1	Female	64	Basal ganglia hemorrhage	VS	MCS+
2	Male	14	TBI	VS	MCS-
3	Male	45	Pontine hemorrhage	VS	eMCS
4	Female	36	Parietal lobe hemorrhage	MCS-	MCS+
5	Male	36	TBI	MCS-	MCS-
6	Male	19	TBI	MCS-	MCS-
7	Female	37	Parietal lobe hemorrhage	MCS-	eMCS
8	Male	46	Basal ganglia hemorrhage	VS	VS
9	Female	32	TBI	VS	eMCS
10	Female	65	Basal ganglia hemorrhage	MCS+	MCS+
11	Female	33	Basal ganglia hemorrhage	MCS-	MCS+

TBI: traumatic brain injuries; VS: vegetative state; MCS: minimum conscious state; eMCS: escape minimum conscious state.

## Data Availability

The data used to support the findings of this study are available from the corresponding author upon request.
